# Syndrome Pattern Recognition Method Using Sensed Patient Data for Neurodegenerative Disease Progression Identification

**DOI:** 10.3390/diagnostics13050887

**Published:** 2023-02-26

**Authors:** Mohd Anjum, Sana Shahab, Yang Yu

**Affiliations:** 1 Department of Computer Engineering, Aligarh Muslim University, Aligarh 202001, India; 2 Department of Business Administration, College of Business Administration, Princess Nourah Bint Abdulrahman University, P.O. Box 84428, Riyadh 11671, Saudi Arabia; 3 Centre for Infrastructure Engineering and Safety (CIES), University of New South Wales, Sydney, NSW 2052, Australia

**Keywords:** neural data, neurodegenerative disease, pattern recognition, recurrent learning

## Abstract

Neurodegenerative diseases are a group of conditions that involve the progressive loss of function of neurons in the brain and spinal cord. These conditions can result in a wide range of symptoms, such as difficulty with movement, speech, and cognition. The causes of neurodegenerative diseases are poorly understood, but many factors are believed to contribute to the development of these conditions. The most important risk factors include ageing, genetics, abnormal medical conditions, toxins, and environmental exposures. A slow decline in visible cognitive functions characterises the progression of these diseases. If left unattended or unnoticed, disease progression can result in serious issues such as the cessation of motor function or even paralysis. Therefore, early recognition of neurodegenerative diseases is becoming increasingly important in modern healthcare. Many sophisticated artificial intelligence technologies are incorporated into modern healthcare systems for the early recognition of these diseases. This research article introduces a Syndrome-dependent Pattern Recognition Method for the early detection and progression monitoring of neurodegenerative diseases. The proposed method determines the variance between normal and abnormal intrinsic neural connectivity data. The observed data is combined with previous and healthy function examination data to identify the variance. In this combined analysis, deep recurrent learning is exploited by tuning the analysis layer based on variance suppressed by identifying normal and abnormal patterns in the combined analysis. This variance from different patterns is recurrently used to train the learning model for maximising of recognition accuracy. The proposed method achieves 16.77% high accuracy, 10.55% high precision, and 7.69% high pattern verification. It reduces the variance and verification time by 12.08% and 12.02%, respectively.

## 1. Introduction

Neurodegenerative diseases (NDDs) are a disorder resulting from the progressive loss of function of selective neurons in the nervous system. As a result, one of the most significant impacts of these diseases is on motor function, which progressively declines [1]. In some cases, the motor function may be affected by paralysis. Therefore, the progressive decline of motor function is one of the major characteristics of neurodegenerative diseases [1]. NDDs are challenging and complex to understand as they involve determining the precise causes of these diseases, identifying optimal approaches for early detection, and developing the most effective treatments. The causes of NDDs are not well understood, but many factors are believed to contribute to the development of these conditions [2]. Some researchers believe genetics play a major role in developing NDDs [3]. However, others believe that environmental factors [1], such as exposure to toxins or traumatic brain injury [4], abnormal medical conditions [5] and ageing [6] contribute to the development pf NDDs. Other concerns are the efficacy of different treatment approaches, with some researchers advocating for more pharmaceutical interventions [7] and others focusing on lifestyle changes [8] and alternative therapies [9]. Many issues exist related to the diagnostic criteria used to identify these diseases. There are established clinical criteria for many NDDs, such as Alzheimer’s disease (AD), Amyotrophic Lateral Sclerosis, and Multiple Sclerosis [10]. Some researchers believe that these criteria are not sensitive enough and may miss early-stage disease [11]. Ongoing research concerns the best methods for tracking disease progression and developing effective treatments [12]. There are also questions regarding the reliability and validity of various diagnostic tools, such as biomarkers and imaging techniques used to track disease progression.

Identifying and monitoring the progression of NDDs is necessary for healthcare systems [2]. Early detection and accurate tracking of NDDs can lead to more effective treatments and improved patient outcomes [11]. It can also help healthcare providers better understand the disease and develop more effective strategies for managing and preventing it [11]. Various techniques and methods are used to identify NDDs [13]. Some of these techniques include brain imaging [14], cerebrospinal fluid analysis [15], genetic testing [16], and cognitive assessments [15]. Each method has its strengths and weaknesses and is often used in combination with others to increase the accuracy and reliability of the diagnosis. Additionally, machine learning (ML) algorithms and artificial intelligence (AI) models are being developed to aid in the identification and progression monitoring of these diseases [11,17]. A monitoring system is used to identify the progression of the NDD by monitoring patients’ conditions and behavioural features. This system detects pathological conditions and collects data necessary for identifying and progressing NDDs. [18]. This system includes monitoring a patient’s cognitive and physical abilities [15], as well as using medical imaging techniques such as magnetic resonance imaging (MRI) or positron emission tomography scans to detect changes in the brain [14]. In addition, biomarkers such as levels of certain proteins or other molecules in the blood or cerebrospinal fluid can be used to indicate the presence or progression of a NDD [15]. A feature space regression model is employed to identify the disease. This model detects spatial and temporal features, increasing clinical accuracy in further processes [19]. Additionally, the model reduces the time and energy consumption in the disease identification process and improves the performance and effectiveness of prediction and identification [19].

Pattern recognition is one of the techniques used to identify specific patterns associated with NDD. By analysing patterns in patient data, such as changes in behaviour, cognitive function, and other physiological factors, healthcare professionals detect the presence and progression of NDD. ML algorithms and other advanced analytical tools are effectively used to help with pattern recognition and the identification of NDD [20]. These algorithms analyse large amounts of data and identify patterns not easily discernible by human observation. These techniques improve the overall accuracy and efficiency of the diagnosis process for NDD [20]. The progression pattern recognition method is utilised to detect NDD by identifying spatial and temporal features of the disease that provide important information for the detection process. To improve the accuracy of predictive models for the risk of NDDs, authors in [20] employ a recurrent neural network with long-short time memory to incorporate temporal information from patients’ medical records into the models. This method involves analysing how a patient’s medical history changes over time and identifying patterns or trends that can be used to predict future disease risks. By incorporating this temporal information, the models can provide more accurate and personalised patient predictions and improve the diagnosis process’s performance [20]. Sequential pattern mining is used to identify relevant patterns and features associated with NDDs [21]. Analysing sequential data collected from individuals over time, such as sensor data, medical records or behavioral data, provides valuable insights into the underlying dynamics of diseases [22]. Disease progression patterns contain important features that provide useful information for detection, prediction and diagnosis. Sequential pattern mining can be used to identify key progressive patterns of behavioural deficits in individuals with NDD [23]. Sequential pattern mining helps improve the diagnosis process’s accuracy by providing supporting evidence for clinical decision-making [24].

ML methods and algorithms are increasingly being used to detect and predict NDDs. These methods effectively analyse patient data, such as medical imaging, genetic data, clinical records, or behavioural data, to identify patterns or features indicative of an NDD [25]. This analysis can include identifying abnormal protein deposits in the brain, changes in brain structure or function, or genetic markers associated with a higher risk of developing NDD [26]. These methods improve the accuracy of pattern recognition, increasing the performance and feasibility of the diagnosis process [25]. These methods have revolutionised the diagnosis and treatment of these diseases in several ways, such as early detection and diagnosis, prediction of disease progression, drug discovery, personalised treatment, monitoring disease progression and treatment effectiveness. Nowadays, deep learning (DL), a subset of ML that involves artificial neural networks, is also increasingly used in the detection and prediction of NDDs [27]. DL algorithms analyse large and complex datasets, such as medical imaging data, to identify subtle patterns or features of these diseases. Convolutional neural networks are the most commonly used algorithm in DL for pattern recognition. These algorithms utilise image data to learn patterns and apply classification techniques to classify patterns based on types and features [28]. They detect spatial and temporal features from the database, maximising the feasibility of the recognition process [29].

Overall, ML methods and algorithms are a promising approach to the detection and prediction of NDDs, and have the potential to improve the accuracy and speed of diagnosis and facilitate the development of new treatments and therapies [30]. Early and precise detection and timely commencement of appropriate therapies, such as medication or behavioral interventions, are critical for improving patient outcomes and quality of life in NDDs [31]. This detection requires an intensive analysis of a range of clinical data, which can be facilitated by data-driven approaches such as ML. This clinical data must be carefully collected and analysed to identify patterns related to NDD [24]. The progression and reversal of disease depend on the underlying cause of the disease, as well as the timing and efficacy of interventions and individual patient characteristics [25,32]. In some cases, interventions may slow or halt disease progression, while in others, interventions may be less effective [25]. Therefore, identifying neurodegenerative disorders often requires the integration of multiple sources of data, including visual and statistical data, as well as input from physicians and other healthcare providers. By combining these different sources of information, it is possible to identify the most recent symptoms and track the progression of the disease through different stages. AI, ML, DL and other data-driven solutions can potentially transform the diagnosis and treatment of NDD by providing more accurate and personalised care [33].

Identifying the progression of NDD is crucial as it can lead to severe consequences if left untreated. Early detection and monitoring of these diseases can help prevent or slow down the progression of symptoms. Therefore, healthcare systems need to have efficient and effective methods for identifying the progression of NDD. By incorporating advanced AI, ML, DL and other data-driven technologies into healthcare systems, early detection can be improved, and the diagnosis and treatment of the disease can be optimised. This research paper presents a novel Syndrome-dependent Pattern Recognition Method (SPRM) for early detection and progression monitoring of NDD, which aims to identify variance between normal and abnormal intrinsic neural connectivity data by using both previously collected data and data from healthy function examination, then combine them and apply deep recurrent learning for analysis of the data. The authors have employed tuning of the analysis layer, based on variations in the data. to suppress and recurrently use those variations to train a learning model for maximising recognition accuracy. This proposed method improves recognition accuracy by suppressing variations in the data. It is a novel approach in the field of NDDs for early detection and progression monitoring using precise identification of physical attributes. Nowadays, these attributes can be easily sensed and observed through wearable sensors. The main contributions of this article are listed below.

1. A pattern recognition method is designed based on disease-specific syndromes to identify their intensity and provide an appropriate diagnosis.
2. Disease progression is identified in the diagnosis stages to improve the medication and reduce unnecessary clinical recommendations in order to retain health stability.
3. Data analysis is performed using different metrics to validate the proposed methods’ consistency and performance.

This research paper is structured in five sections, starting with an introduction in Section 1, which provides background information and sets the context for the research. In Section 2, the paper presents an overview of related work in the field, discussing previous methods and studies that have been conducted on the topic. Section 3 presents the proposed SPRM, describing the method in detail and explaining its key features. Section 4 provides the results and discussion, demonstrating accuracy, precision, pattern verification, variance, verification time, analysis of the method’s performance, and discussion of its results. Finally, in Section 5, the paper concludes by summarising the main findings and contributions of the research.

## 2. Related Work

Recently, there has been a growing interest in NDD identification and progression. Various methods and techniques have been proposed in the literature to improve the accuracy and efficiency of the diagnosis process. This focus is driven by the need to improve the diagnosis and treatment of NDD, which can have severe and debilitating effects on patients if left untreated. Some of the research in this area has focused on using advanced techniques such as AI, ML, DL and other data-driven models to analyse large amounts of data and identify patterns and features indicative of disease progression. Other research has aimed to develop new imaging techniques and biomarkers that can help detect these diseases early. Some recent research works propose and implement sophisticated AI, ML, DL and other data-driven models for NDD identification and progression. These works are explained as follows.

Amyotrophic Lateral Sclerosis is a devastating NDD with no cure, which causes rapid degeneration of motor neurons and can result in death by respiratory failure. Non-invasive Ventilation is an effective treatment that can prolong survival and improve quality of life. Predicting the need for Non-invasive Ventilation is crucial for timely administration and better patient outcomes. In [34], the authors applied itemset and sequential pattern mining to identify disease presentation and progression patterns, respectively, and trained the prognostic models that incorporate static and temporal features. The case study outcomes showed promising results, with bulbar function, phrenic nerve response amplitude, and respiratory function identified as significant features. These findings align with clinical knowledge regarding relevant biomarkers of disease progression towards respiratory insufficiency. Predicting the long-term progression of AD is also a crucial aspect of disease management. The literature analysis uncovers that the existing methods have focused on predicting cognitive scores. Therefore, Zhao et al. [35] proposed a framework that used a 3D multi-information generative adversarial network to predict an individual’s whole brain appearance at future time-points, along with a 3D DenseNet-based multi-class classification network to determine the clinical stage of the estimated brain. The results show that the proposed framework outperforms the existing methods, with a high structural similarity index between the generated and real MRI images, and the use of focal loss improves accuracy in determining the clinical stage. The proposed framework has the potential to provide more information for accurate long-term disease progression prediction and, ultimately, to improve AD patient management.

Peng et al. [36] proposed white-matter features from positron-emission tomography-based progression for mild cognitive impairment to AD. The proposed approach involves an ML model for detecting disease progression and utilises multivariate logistic regression to assess the relevant characteristics and features of the detection process. The proposed model predicts the white matter changes in the brain, reducing the error rate in diagnosis and identification processes. Johnson et al. [37] developed a multi-modal quantitative approach (MMQA) for predicting the progression of NDD. The proposed multimodal identifies key anatomical and metabolic changes that correlate with the progression of pathological and behavioural deficits in NDDs. By monitoring 144 parameters longitudinally using non-invasive neuroimaging modalities and kinematic gait analysis, the researchers developed a highly sensitive platform that can be used for preclinical studies. The results of this study suggest that this approach has the potential to be a powerful tool for clinicians in the future, providing valuable insights into the progression of NDDs. De Vos et al. [38] introduced an ML-based method for detecting progressive supranuclear palsy using random forest and logistic regression algorithms. The proposed method also distinguished progressive supranuclear palsy from Parkinson’s disease (PD) by classifying patterns based on specific conditions. An array of wearable sensors was used to create the dataset for the ML model. The introduced method improves the overall accuracy of progressive supranuclear palsy detection, increasing the effectiveness and reliability of the system.

Kmetzsch et al. [39] proposed a new framework for computing a disease progression score from cross-sectional multimodal data. A supervised multimodal variational autoencoder was used to infer a meaningful latent space, where latent representations were placed along a disease trajectory, and orthogonal projections computed a score onto this path. The framework was evaluated with multiple synthetic and real datasets, and results demonstrated better performance than state-of-the-art approaches. The proposed framework can objectively measure disease progression with potential applications in clinical trials. Zhao et al. [40] designed a multimodal gait recognition for NDDs (MGR-ND). The proposed novel hybrid model learnt gait differences between three NDDs, PD severity levels, and healthy individuals. The model fused and aggregated data from multiple sensors and applied a spatial feature extractor and a new correlative memory neural network architecture to capture temporal information. A multi-switch discriminator was then used to associate observations with individual state estimations. The proposed framework outperformed several state-of-the-art techniques in classification accuracy. Alorf et al. [41] presented a new approach to the multi-label classification of AD’s stages using resting-state functional MRI and deep learning. The proposed model extracted the brain’s functional connectivity networks from resting-state functional MRI data and utilised Stacked Sparse Autoencoder and Brain Connectivity Graph Convolutional Network deep learning approaches to solve the multi-class classification problem. The proposed models achieved an average accuracy of 77.13% and 84.03% for multi-label classification using Stacked Sparse Autoencoders and Brain Connectivity Based Convolutional Networks, respectively. The study also identified significant brain regions of interest by analysing the network’s learned weights.

Dentamaro et al. [42] developed a method for discriminating NDD patterns by analysing human gait with 2D cameras. The proposed method used the kinematic theory of rapid human movements and other spatiotemporal features to model the human gait movement pattern. The results demonstrated the effectiveness of this approach in describing neurodegenerative patterns, achieving 99.1% accuracy when used in conjunction with state-of-the-art pose estimation and feature extraction techniques. In [43], an AI and wavelet coherence (AI-WC) based model was proposed. This model comprised a convolutional neural network and wavelet coherence spectrogram of gait synchronisation to classify NDDs based on gait force signals. The algorithm was evaluated using an existing online database, and the results showed that the proposed method effectively differentiates gait patterns between healthy control and NDD patients, with an overall sensitivity of 94.34%, specificity of 96.98%, the accuracy of 96.37%, and AUC value of 0.97 using 5-fold cross-validation. The proposed algorithm has the potential to aid physicians with screening for NDDs for early diagnosis, efficient treatment planning, and monitoring of disease progression. Lei et al. [44] implemented an adaptive feature learning framework using multiple templates for the early diagnosis of NDDs. The proposed method was validated on AD and PD databases and outperformed the state-of-the-art methods. Different features were extracted and fused, and a feature selection was applied with an adaptively chosen sparse degree. In addition, linear discriminative analysis and locally preserving projections were integrated to construct a least square regression model. The proposed method demonstrated that accurate feature learning facilitates the identification of highly relevant brain regions with significant contributions to the prediction of disease progression.

Bi et al. [45] established a knowledge base to systematically understand the heterogeneity of the risk factors associated with different NDDs, which they refer to as pan-NDDs. This knowledge base aims to facilitate personalised and knowledge-guided diagnosis, prevention, and prediction of NDDs. The authors outlined the knowledge base’s structure and content, including information on the epidemiology, genetics, environmental and lifestyle factors, clinical and neuropathological features, and treatment options for NDDs. The potential applications of the knowledge base are also discussed, including its use in clinical decision-making, drug development, and public health policies. Overall, the knowledge base is intended to provide a valuable resource for researchers, clinicians, and patients in the field of NDDs. Beyrami et al. [46] proposed a new approach based on statistical and entropic features of vertical ground reaction forces of gait and sparse coding classification techniques. The study explored the effect of individual differences on the proposed and standard ML methods, emphasising the severity and duration of diseases and the right and left foot parameters. The study results indicated that, using left or right foot features, the proposed algorithm could identify all NDDs at early and advanced stages. Van Veen et al. [47] used F-fluorodeoxyglucose positron emission tomography and Principal Component Analysis to identify disease-related brain patterns in neurodegenerative disorders. Nevertheless, they found that Principal Component Analysis has limitations in discriminating between different conditions. To overcome this, Generalized Matrix Learning Vector Quantization was applied to F-fluorodeoxyglucose positron emission tomography scans of healthy controls and patients with AD, PD, and Dementia with Lewy Bodies. The study demonstrated that Generalised Matrix Learning Vector Quantization is a more advanced ML algorithm that can provide a solution to discriminate between different neurodegenerative conditions.

The literature analysis shows that recent research in neurodegenerative disease identification and progression has focused on developing sophisticated AI and ML models and algorithms that can accurately and efficiently detect, track and predict the progression of these diseases. Some examples include developing models that can predict the need for non-invasive Ventilation and using ML to classify patterns and features of the disease. Researchers have also been exploring the use of multimodal approaches that combine multiple imaging modalities, such as MRI, functional MRI and positron emission tomography, to gather more comprehensive information for diagnosis and classification. Various algorithms, such as random forest, logistic regression, and generative adversarial networks, have been used to classify the patterns and improve the diagnosis process.

## 3. Proposed Syndrome-Dependent Pattern Recognition Method

**Figure 1:**
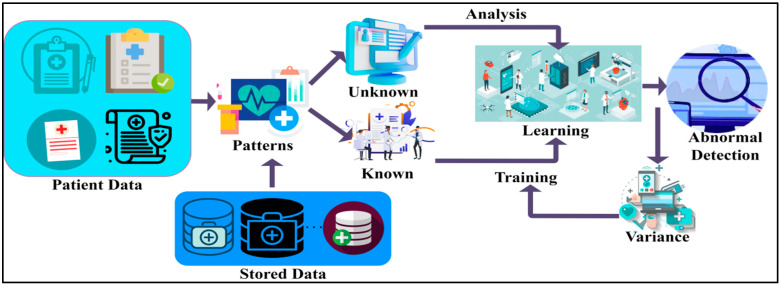
The proposed Syndrome-dependent Pattern Recognition Model.

This research article introduces an SPRM for the early and progressive detection of NDD. This method determines the variance between normal and abnormal intrinsic neural connectivity data. Pattern recognition also helps classify unknown data, improves the accuracy of predictions, and allows for the identification of learning techniques. This method can also generate predictions for unknown data and helps in practical decision-making. It can acknowledge and associate an object at various distances. The proposed method utilises recurrent learning, a commonly used method for handling sequential data before developing attention models. Recurrent learning is used to predict the problems in the method and recognise the speech that may be given as the input in the method. The workflow diagram of the proposed model is presented in Figure 1.

Here in this method, the patient data is given as the input to recognise the patterns. Then it is checked with the data already stored. After identifying the patterns of the neural data, it is classified as unknown data or normal data. Unknown data is that found anew, without matching the existing data. Then, the acquired normal data is that which retains the previous value of the patients, and matches the previous data. It can be used as input for the training process. The results are analysed from the received unknown data using deep recurrent learning. This can also help recognise the data pattern by using the learning technique to identify the results. It can help generate divinations of unknown data and helps in preparing practical decisions. This combined analysis exploits deep recurrent learning by tuning the analysis layer based on variance. The variance is suppressed by identifying normal and abnormal patterns in the combined analysis. Variance can be both high and low, depending on the data pattern, to determine the accuracy of the patients’ disease level. If variance occurs, then separate training will be given with the normal data pattern to reduce the variance. This variance from different patterns is recurrently used for training the learning model to maximise recognition accuracy. The variance is the difference from the previously acquired data. If there is an increase in the variance, then the intensity of the neurodegenerative disease should be identified with the recognised data pattern. The output of the variance is represented as the progression. From this output, the abnormality of the patient’s disease can also be recognised, and processes can be carried out to reduce the abnormality. Abnormal results are those that are determined from the unknown data from the data pattern. The patients’ data, which is observed at different times, is given as the input for the process to recognise the data pattern. The process of fixing the patient data, which is observed at different times as the input for the further procedure, is explained by the following Equation (1):

(1) $$A_{\alpha }=\{\begin{array}{ll} \;0, & \;if\;\alpha =0\\ \sigma \left(A_{\alpha -1},X_{\alpha }\right), & \;otherwise \end{array}$$

where ($A_{\alpha }$) is denoted as the patient’s data which is represented as the input, ($X_{\alpha }$) is denoted as the observation time, and (σ) is denoted as the calculation of the data in different observation times. Now the patient’s data input is sent to recognise the pattern. The patient data is given as the input to recognise the pattern. Then it will be checked with the already stored data. The input neural data is sent to the pattern recognition process to identify whether it is unknown data or normal. The given input is checked with the stored data of the patient. This process is used to investigate whether the given input data matched the existing data of the patients. This pattern recognition is used to predict the present state of the patient’s disease, and to identify the variance which results in progression.

The recognition of the neural data is used to check the availability of the data which is already stored with the information. The input patient data is checked with the existing stored data to determine the matching. The input given is observed at different times and sent to settle the data pattern. This data pattern recognition is used to identify the difference between normal and abnormal connectivity. The already stored data contains the exact information about the patient’s disease and the state of the disease. By checking with the stored data, the similarity of the input data can be established. After recognising the patterns of the neural data, it is classified as unknown data or normal data. It is also used to verify the pattern of the data. After recognising the data pattern of the patients’ neurodegenerative disease, it can be classified into two types: unknown data, determined newly, and normal data, which match the stored data. This technique is used to determine the accuracy of the disease. The process of recognising the data pattern is explained by the following Equation (2):

(2) $$A_{\sigma }=\sigma \left(WX_{\alpha }+SA_{\alpha -1}\right)$$

where (W) is denoted as the process of data pattern recognition, and (S) is denoted as the existing stored data of the patients. Now, the data pattern recognising process identifies the unknown and normal data. The unknown data which is acquired from the process of data pattern recognition is the one that is newly received without any matching with the existing stored data. This is the data pattern recognised newly from the patient’s disease. After recognising the patterns of the neural data, it is classified as unknown data or normal data. Unknown data is that which is found anew, without matching the existing data. The previous data does not match the acquired data. These are the data determined newly after the process of data pattern recognition. This process results in obtaining the unknown data pattern from the patients’ neural data. This results in analysis of the unknown results by using recurrent learning to identify the progression results of the disease.

**Figure 2:**
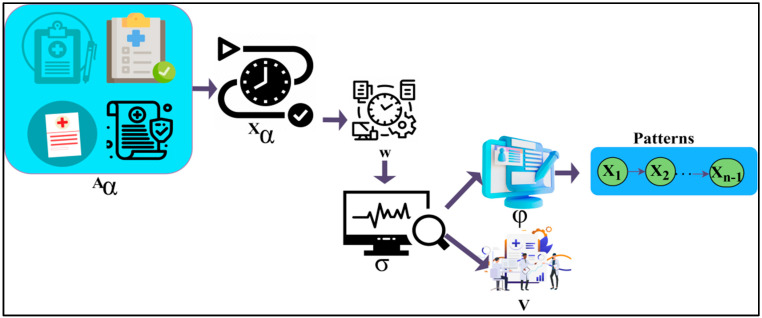
Schematic diagram of data recognition.

After the process of data recognition, the data are categorised into unknown data and normal data. The unknown data do not match the stored data of the patients. Those data are observed at different times. This method is used to determine the variance that appeared in the training period by using the normal data, which matches the existing stored data. These data produce abnormal detection of the patients’ neurodegenerative disease and produce unknown results. This will not be similar to the state of the stored patients’ data and does not match those values of the disease. These unknown data are used in the procedure of analysing the operation, whereas normal data is used in training to deliver the perfect progression result concerning the patient’s disease and its state. The results are analysed from the received unknown data using deep recurrent learning. It can also be helpful in recognising the data pattern to identify the results using the learning technique. The observed data is combined with previous and healthy function examination data to identify the variances. The process of determining the unknown data from the data pattern recognition procedure is explained by the following Equation (3):

(3) $$Q\left(X_{1},X_{2},\ldots .X_{n}\right)D(X_{1})\ldots D\left(X_{n}|X_{1}\ldots .X_{n-1}\right)$$

where (Q) is denoted as the unknown data, and (D) is denoted as the unknown data pattern of the acquired data. After recognising the patterns of the neural data, it is classified as unknown data or normal data. The patient data is given as the input to recognise the pattern. Then it will be checked with the already stored data. The data pattern recognition process results show that normal data can be determined. Then the acquired normal data is that which retains the previous value of the patients, which matches the previous data. It can be used as input for the training process. These data retain the previous value of the previous patients’ neural disease reports. These match the already existing stored data of the patient. These data can be used as the input for the training. This can help detect the variance between the connectivity. The schematic diagram of data recognition from the distinguishable data (patterns) is presented in Figure 2.

The sequence $\forall \;X_{1}$ to $X_{n-1}$ (leaving out the next W) from $A_{\alpha }$ is observed through $X_{\alpha }$. In this observation instance, the σ∈W is classified for φ and D for preventing V overlaps. The normal data is recognised from the data pattern recognition procedure. This is the one which matches the stored data already existing in the process. This procedure has complete information on patients’ NDD, as shown in Figure 2. The process of acquiring the normal data from the data pattern recognition process is explained by the following Equation (4):

(4) $$V\;X_{n}|X_{1},\ldots .X_{\alpha -1})=\varphi \left(A_{\alpha }\right)$$

where (V) is denoted as the normal data obtained from the data pattern recognition process, and ($\;\varphi \left(A_{\alpha }\right)$) is denoted as the calculation of the similarities of the found data with the stored data of the patients’ neural diseases. Now the normal data is used as the input of the training to detect the variance. The unknown data was used for the analysis process by using deep recurrent learning. It can also be helpful in recognising the data pattern to identify the results by using the learning technique. It generates divinations of unknown data and helps in preparing practical decisions. The recurrent learning technique is used to predict decisions concerning the accuracy of the connectivity. It is also used to determine the abnormality of the disease by using unknown data from the data pattern recognition process. This analysis process helps to identify the unknown results which are produced by the unknown data from the process. It does not match any of the stored data, and it does not retain the observed values of the patients. This learning is used to predict the problems in the method and gives perfect solutions to resolve those abnormalities. This combined analysis exploits deep recurrent learning by tuning the analysis layer based on variance. From the training process, the variance is detected with the help of the normal data. Variance is the difference between the previous data and the present data. The process of analysis by using deep recurrent learning is explained by the following Equation (5) [34]:

(5) $$\begin{array}{c} A_{\alpha }=B_{\alpha }\Delta \;\left(C_{\alpha }\right)\\ B_{\alpha }=\theta \;\left(W_{oi\;}X_{\alpha }+W_{oA}\;A_{\alpha -1}+W_{oC}\;C_{\alpha }\right)\\ \begin{array}{c} C_{\alpha }=i_{\alpha }\forall {\hat{C}}_{\alpha }+D_{\alpha }\forall C_{\alpha -1}\\ D_{\alpha }=\theta \;\left(\;W_{Fi}X_{\alpha }+W_{FA}A_{\alpha -1}+W_{FC}C_{\alpha -1}\;\right) \end{array} \end{array}\}$$

where ($B_{\alpha }$, $D_{\alpha },\;C_{\alpha }$) is denoted as the analysing process with the help of the unknown data which produces the unknown results, (θ) is denoted as the dissimilar data acquired, (F, i) is denoted as the process of combined analysis, and (∀) is denoted as the values of the disease. Now the normal data is used as the training input, which helps detect the variance. Variance is the difference occurring between the previous and the acquired data. This learning technique is used to determine the high variance and the low variance. This combined analysis exploits deep recurrent learning by tuning the analysis layer based on variance. Variance can be both high and low, depending on the data pattern, to determine the accuracy of the patients’ disease level.

**Figure 3:**
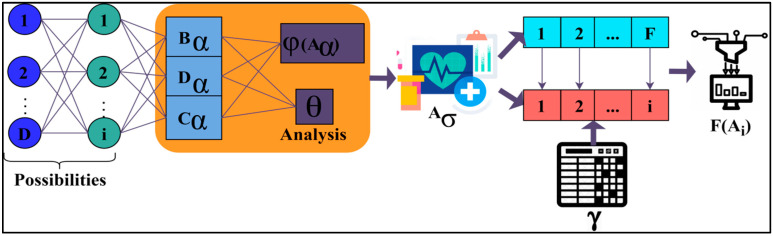
Combined analysis process.

If variance occurs, then separate training will be given with the normal data pattern to reduce the variance. The occurrence of variance causes the abnormal detection of the patient’s disease and needs separate training to resolve the abnormalities. It can produce both a high and low variance depending on the state of the patient’s disease. It also identifies abnormalities if they occur in the process of detecting the accuracy of the disease. This variance from different patterns is recurrently used for training the learning model for maximising recognition accuracy. The variance is the difference from the previously acquired data. The process of detecting the variance from the analysis procedure by using the unknown data and normal data in training is explained by the following Equations (6) and (7):

(6) $$F\left(A_{i}\right)=\{\begin{array}{c} \forall \left(A_{i}\right),\;\;\;if\;A_{i}>0\\ \gamma_{i}\forall \left(A_{i}\right),\;\;\;if\;A_{i}\leq 0 \end{array}$$

(7) $$F\left(A_{i}\right)=\begin{array}{c} \lambda (0,\;F\left(A_{i}\right)\\ \lambda (0,\forall \left(A_{i}\right) \end{array}\}$$

where ($F\left(A_{i}\right)$) is denoted as the process of determining the variance, and (λ) is denoted as the connectivity. From the result of the variance, high and low variances can be found. If there is an increasing order of variance, then the intensity of the disease should be identified to eliminate the abnormalities. This is the divergence between the present and the existing data. It is used to combine the observed data with the previous healthy functions to detect the variances in the process and further steps provided to reduce the variance. Separate training is given to reduce the variance with the help of the normal data, which is given as the input. The combined analysis based on the similarity process is displayed in Figure 3.

**Figure 4:**
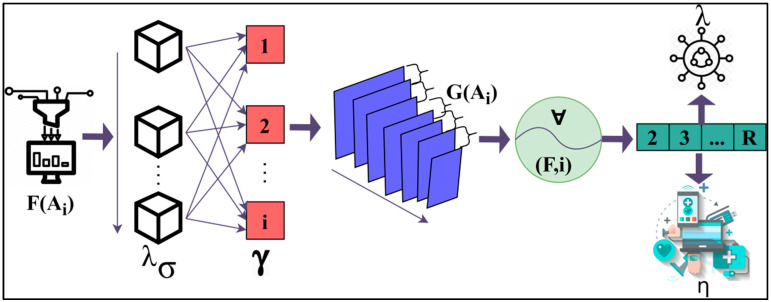
Learning for abnormalities.

The variance can be high or low according to the similarities of the data. Then separate training is given to reduce the variance with the help of the normal data, which is given as the input of the training. Based on this variance, the analysis process is done by using deep recurrent learning. This helps in identifying the intrinsic connectivity variances under two-layers. In the first layer, the possibilities for D and i are extracted for checking $A_{\alpha }$ and θ combinations. These two processes are consequent such that the learning process discussed above is analysed using two layers, one for variance and the other for λ estimation, as depicted in Figure 3. The first layer defines the $F\left(A_{i}\right)$ for different variances; the next function is the θ for identifying similarity. This function is different from the previous layer by identifying the remaining iterations and distinguishable F. The learning process is deployed for classifying normal and abnormal variances from the observed data. This classification is performed to prevent unidentified data features from influencing the analysis process without increasing the variance. The process of finding the variance between the normal and abnormal neural data connectivity is explained by the following Equations (8)–(10) [23]:

(8) $$G\left(A_{i}\right)=\lambda \left(0,\;\forall \left(A_{i}\right)\right)+\gamma_{i}\lambda_{\sigma }\left(0,\forall \left(A_{i}\right)\right)$$

(9) $$\frac{\partial R}{\partial \gamma_{i}}=\underset{A_{i}}{\sum }\frac{\partial L}{\partial F\left(A_{i}\right)}\frac{\partial F\left(A_{i}\right)}{\partial \gamma_{i}}$$

(10) $$\frac{\partial F\left(A_{i}\right)}{\partial G\lambda_{i}}=\{\begin{array}{c} 0,\;\;\;\;\;if\;A_{i}>0\\ I\left(A_{i}\right),\;\;\;\;\;if\;A_{i}\leq 0 \end{array}$$

where ($G\left(A_{i}\right)$) is denoted as the process of finding the variance between the neural data connectivity, and ($\frac{\partial R}{\partial \gamma_{i}}$) is denoted as the process of determining the observed data combined with the health function examination data. Now, from the variance output, the abnormality can be identified and resolved. The observed data is combined with previous and healthy function examination data to identify the variances. This combined analysis exploits deep recurrent learning by tuning the analysis layer based on variance. In Figure 4, the abnormality detection using the learning process is presented.

**Figure 5:**
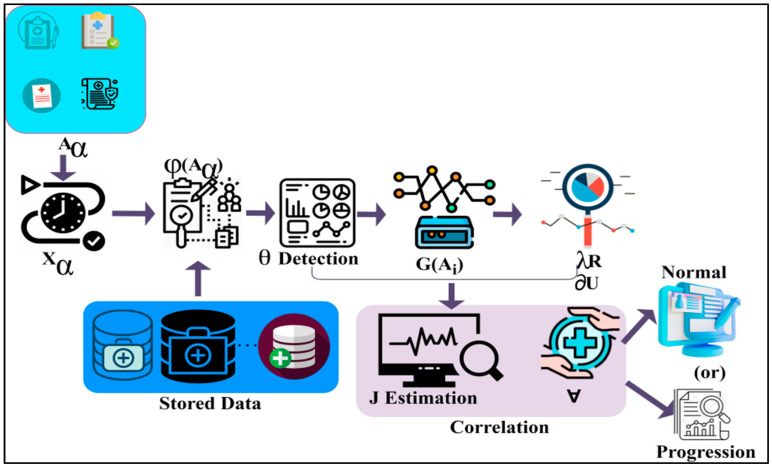
The variance-based progression detection process.

The λ is estimated between two sequences from the second R for ∀ ∈(F,i). In the $G\left(A_{i}\right)$ extraction the R segregates λ and η ∀ i from $\lambda_{\sigma }$. In the $\gamma *\;\lambda_{\sigma }$ assessment of the input $F\left(A_{i}\right)$ identifies abnormalities through recurrent iterations, as displayed in Figure 4. The abnormalities are found by the unknown data and results which do not match the previous data stored. With the help of the variance, the accuracy of the disease state and the abnormalities that occurred in it can be identified. Further steps such as more training can be performed to reduce the abnormalities. The process of acquiring the abnormality from the output of the variance can be explained by the following Equation (11):

(11) $$\frac{\partial R}{\partial U}=\underset{i}{\sum }\underset{u_{i}}{\sum }\frac{\partial R}{\partial U\left(A_{i}\right)}\frac{\partial F\left(A_{i}\right)}{\partial \gamma }$$

where ($\frac{\partial R}{\partial U}$) is denoted as obtaining the abnormality from the output of the analysing process and variance. Now, the progress report of the situation of the patient’s disease is made by the output of the variance between the normal and abnormal intrinsic neural data connectivity. If the progression is abnormal, further steps are taken to reduce the abnormalities in the patient’s disease report. The process of providing the progression report by the output of the variance is explained by the following Equations (12)–(14):

(12) $$ŋ\gamma_{i}=\lambda ŋ\gamma_{i}+\underset{ŋ\gamma_{i}}{\sum }\frac{\partial R}{\partial \gamma_{i}}$$

(13) $$\hat{A_{i}}=\frac{A_{i}-ŋ\gamma_{i}J\left[A_{i}\right]}{\sqrt{Z\left[A_{i}\right]}}$$

(14) $$Z\left[A_{i}\right]=a_{i}\hat{\Delta_{i}}+J\left[A_{i}\right]$$

where ($ŋ\gamma_{i}$) is denoted as the output of the variance detecting process, (J) is denoted as the intrinsic neural data connectivity, and ($Z\left[A_{i}\right]$) is denoted as the process of determining the progress report of the patients’ neurodegenerative disease. The progression detection using the variance is presented in Figure 5.

The progression is extracted by correlating J and ∀ from the $G\left(A_{i}\right)$ estimation. Considering the $\varphi \left(A_{\alpha }\right)$ and θ between $A_{\alpha }$ and $\frac{\partial R}{\partial U}$, the variance is computed. The learning segregates the V and λ for ease of progression detection. Compared to the available stored data, if ∀ varies to an extreme value, then progression is measured, as shown in Figure 5. 

This research article discussed SPRM for the early and progression detection of NDD. Pattern recognition also helps in classifying unknown data. It makes valuable predictions and identifies the learning techniques. The observed data is combined with previous and healthy function examination data to identify the variances. This combined analysis exploits deep recurrent learning by tuning the analysis layer based on variance. This variance from different patterns is recurrently used for training the learning model to maximise recognition accuracy.

## 4. Results and Discussion

This section is divided into model analysis and comparative analysis to illustrate the quantitative work using the dataset. The self-analysis involves analyzing the matching features extracted using the clinical observation discussed in the proposed method. This real-time data analysis is performed using the representation and the observation sequences. The patterns observed in the sequences are correlated with the proposal for verifying its efficiency and validating the statistical performance. In contrast, the comparative analysis continues the self-analysis and the representations depicted in the subsequent subsections. Besides self-consistency, out-of-box verification is required to prove the stability of the proposed concept. Therefore, the results associated with the data features are comparatively analyzed. Alongside the metrics, the process features such as time, variances observed, and their impact on the proposed method are elaborated.

### 4.1. Dataset Description and Model Analysis

**Figure 6:**
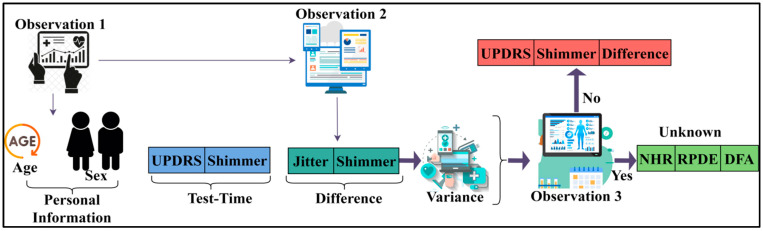
Unknown sequence pattern features.

The analysis for identifying disease prediction is performed using PD progression data [48]. This source provides observed information from 42 human subjects for detecting PD progression. A total of 16 fields correlating personal and medical information are recorded for the corresponding progression detection. The motor operations, harmonics, fluctuation, entropy, jitter, testing time, etc., are the features used for detecting progression. A total of 5876 records are used for analysis of the patterns and progression. The progression is observed through 120–148 sensing instances at different intervals. These patterns for the known and unknown sequences are extracted as presented in Figure 6.

**Figure 7:**
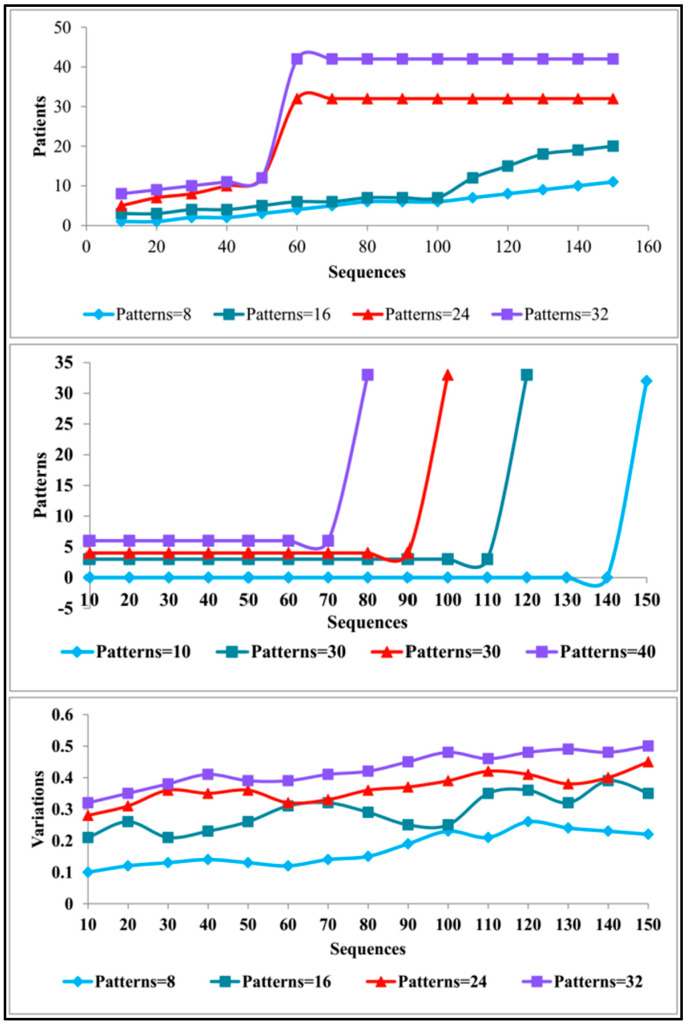
Observation analysis.

The sequence variance determines its need for unknown detection. The observations identify Unified Parkinson’s Disease Rating Scale (UPDRS) and shimmer during the first and normal test times. Contrarily, if a difference due to fitter and shimmer is observed, then it is a variance. This requires Noise-to-Harmonics Ratio (NHR), Recurrence Period Density Entropy (RPDE), and Detrended Fluctuation Analysis (DFA) observations (additional) during the next sequence. In this case, the unknown pattern features are identified in the (next) successive observation, as depicted in Figure 6. The variance is estimated using different ranges as defined by the disease correlation values. Say, for example, the DA (different amplitude) between two successive sequences ranges between 0.4 and 0.6. The exceeding range (beyond 0.6) is termed a variance. Therefore, the “Jitter” and “Shimmer” above 0.6 is marked as unknown. The additional NHR, RPDE, and DFA are observed to prevent disease progression detection errors. Therefore, the number of additional observations required among the 42 patients between 120 and 148 sequences is presented in Figure 7.

**Figure 8:**
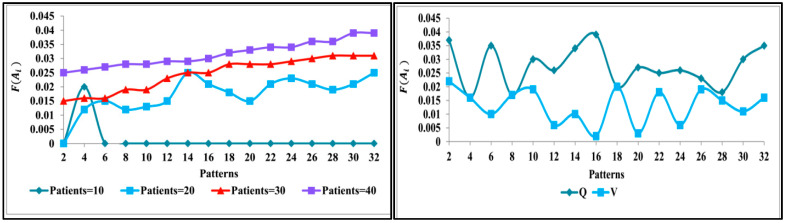
$F\left(A_{i}\right)$ analysis.

The observation of patterns and variations for different sequences is presented in Figure 7. The pattern across different observations is validated if any unknown information is sensed. Therefore, the analysis is performed to extract abnormalities under varying sequences. Hence, the consecutive training iteration relies on analysis other than classification. This is validated until NHR (or) RPDE (or) DFA clarifies the patterns from the observed patient data. The intense assessment is concluded if the variance (between sequences) is stabilised. The variance achieves its maximum output without increasing/decreasing, as interpreted from Figure 7. The variations are identified from the φ and V patterns based on λ for which $G\left(A_{i}\right)$ is computed. The variance for progression estimation is set as 0.06 (from the Jitter RPP) (max), and therefore the decision is performed. This average progression value varies with the patient’s physical attributes (age, disorder, healthy level, etc.). The different (mean) variation across the different patterns is analysed in Figure 8.

**Figure 9:**
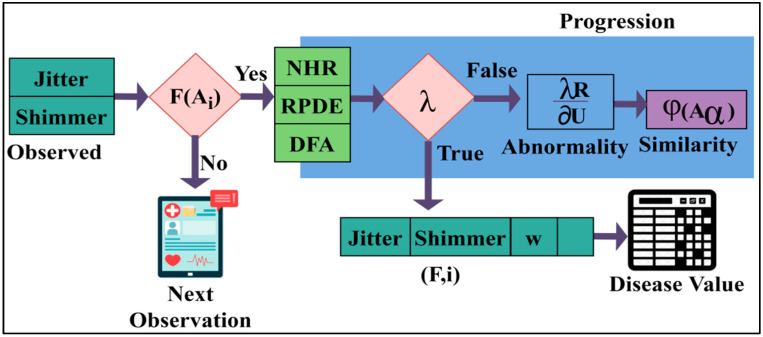
Progression classification.

The $F\left(A_{i}\right)$ analysis is presented in Figure 8 for the varying patterns. This analysis considers the varying patients and (Q,V) depending on the λ. The λ for the observable $\rho \left(A_{\alpha }\right)$ achieves less $f\left(A_{i}\right)$; this is true under less available patient data. Contrarily, if $\frac{\partial R}{\partial r_{i}}$ is required variation (function verification), then the $F\left(A_{i}\right)$ increases such that $X_{\alpha }$ requires a new instance. Therefore, the $\frac{\partial R}{\partial U}$ is the consecutive derivative function for abnormality detection. In this process, the learning process identifies the $F\left(A_{i}\right)$ suppression condition for maximising precise $Z\left(A_{i}\right)$. Now, the progression classification based on D is performed as presented in Figure 9.

The progression is analysed from $F\left(A_{i}\right)=true$ condition till a $\rho \left(A_{\alpha }\right)$ is observed. Therefore $Z\left[A_{i}\right]$ is the combination of (F,i) and $G\left(A_{i}\right)$ between two consecutives λ. Hence a new observation is required to prevent false progression detection. Considering the differences across various $X_{\alpha }$, the θ and $\frac{\partial R}{\partial r_{i}}$ are utilised for λ verification and $\eta r_{i}$ assessment, as shown in Figure 9.

### 4.2. Comparative Analysis

This section presents the discussion of comparative analysis by performing comparison of proposed SPRM with the existing methods—MMQA [37], MGR-ND [40], and AI-WC [43]. This analysis computes the performance matrices’ accuracy, precision, pattern verification, variance, and verification time. From the data source, the inputs are varied from 500 to 5000, and the patterns are varied from 2 to 32.

#### 4.2.1. Accuracy

**Figure 10:**
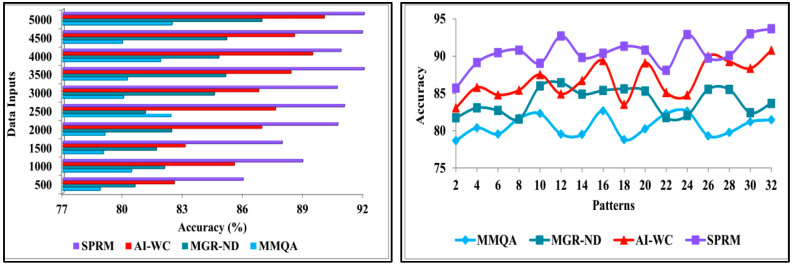
Accuracy analysis.

The accuracy of the recognition process is efficacious in this method by using the SPRM. After recognising the patterns of the neural data, it is classified as unknown data or normal data. The results are analysed using deep recurrent learning from the unknown data. It can also help recognise the data pattern to identify the results by using the learning technique. It generates divinations of unknown data and helps in preparing practical decisions. This method identifies the variance between normal and abnormal intrinsic neural connectivity data. The variance is the difference from the previously acquired data. If there is an increase in the variance, then the intensity of the neurodegenerative disease should be identified with the recognised data pattern. The output of the variance is represented as the progression. From this output, the patient’s disease’s abnormality can also be recognised, and processes can be carried out to reduce the abnormality. Through this process, the accuracy of the recognition is increased. Figure 10 depicts the comparison of accuracy for implemented SPRM, and existing MMQA, MGR-ND, and AI-WC for different data inputs and patterns.

#### 4.2.2. Precision

**Figure 11:**
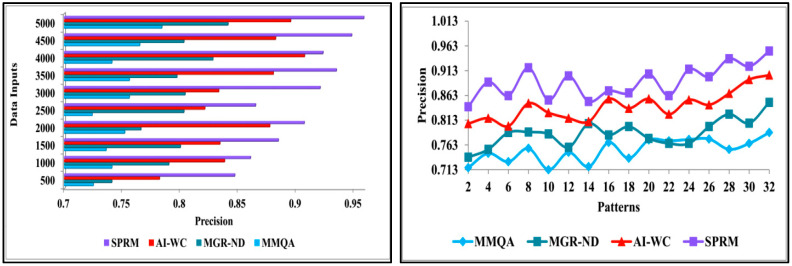
Precision analysis.

The precision is high in this process using the SPRM and the deep recurrent learning technique. At first, the data pattern is recognised with precision, and then it is classified into unknown data and normal data. It is determined by the patients’ neural data, which is observed at different times. This method identifies the variances in different observation intervals. The observed data is combined with previous and healthy function examination data to identify the variances. In this combined analysis, deep recurrent learning is exploited by tuning the analysis layer based on variance. The variance is suppressed by identifying normal and abnormal patterns in the combined analysis. This is the divergence between the present and the existing data. It is used to combine the observed data with the previous healthy functions to detect the variances. Based on this variance, the analysis process is carried out by using deep recurrent learning. This helps in identifying the intrinsic connectivity using the variances in each observation instance. Figure 11 shows the comparison of precision for implemented SPRM, and existing MMQA, MGR-ND, and AI-WC for different data inputs and patterns.

#### 4.2.3. Pattern Verification

**Figure 12:**
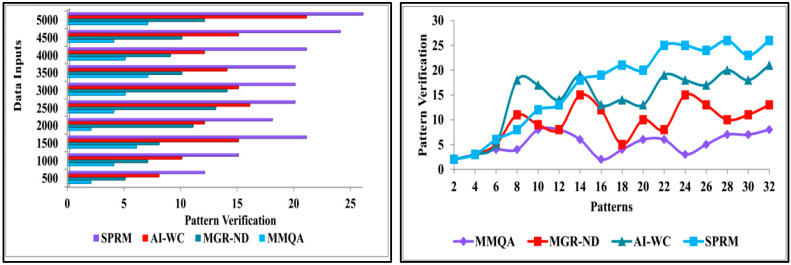
Pattern verification analysis.

The pattern verification is highly accurate using the SPRM and the recurrent learning technique. The patient data is given as the input to recognise the pattern. Then, it will be checked with the already stored data. Based on the data features and variances, the progression is identified in this proposed method. The variances indicate the chances of the risks by estimating precise patient behavior. This behavior varies with the actual body conditions of either risk or nil risks. The input neural data is sent to the pattern recognition process to identify whether it is unknown data or normal. The given input is checked with the stored data of the patient. This process is used to investigate whether the given input data matched the existing data of the patients. This pattern recognition predicts the present state of the patient’s disease. This is also used to identify the variance which results in progression. By checking with the stored data, the similarity of the input data can be established. After recognising the patterns of the neural data, it is classified as unknown or normal data. It is also used to verify the pattern of the data. After recognising the data pattern of the patients’ neurodegenerative disease, it can be classified into two types: unknown data, which is determined newly, and normal data, which matches the stored data. Figure 12 displays the comparison of pattern verification for implemented SPRM, and existing MMQA, MGR-ND, and AI-WC for different data inputs and patterns.

#### 4.2.4. Variance

**Figure 13:**
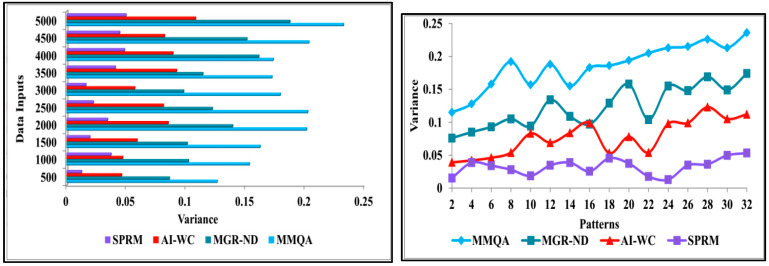
Variance analysis.

The occurrence of variance is less in this process using deep recurrent learning. The normal data is used as the input to the training process, which helps in detecting the variance. Variance is the difference that occurs between the previous and the acquired data. This method identifies the different variances across distinguishable patterns. This learning technique is used to determine the high variance and the low variance. This combined analysis exploits deep recurrent learning by tuning the analysis layer based on variance. Variance can be both high and low depending on the pattern of the data in determining the accuracy of the patients’ disease level. If variance occurs, then separate training will be given with the normal data pattern to reduce the variance. The occurrence of variance causes the abnormal detection of the patient’s disease, and this needs extra separate training to resolve the abnormalities. This variance from different patterns is recurrently used for training the learning model to maximise recognition accuracy. Figure 13 shows the comparison of variance for implemented SPRM, and existing MMQA, MGR-ND, and AI-WC for different data inputs and patterns.

#### 4.2.5. Verification Time

**Figure 14:**
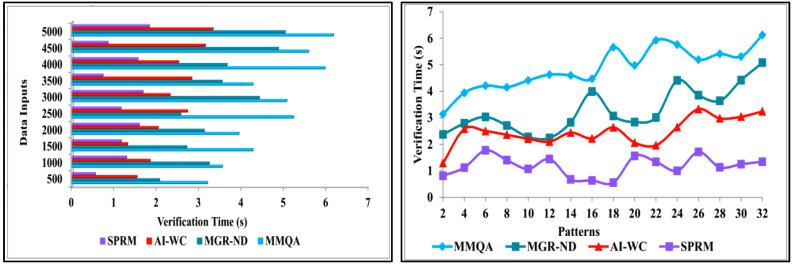
Verification time analysis.

Data pattern verification and variance occurrence are less in this method using SPRM and learning techniques. This method identifies the variance between normal and abnormal intrinsic neural connectivity data. This learning technique is used to determine the high variance and the low variance. Based on this variance, the analysis process is carried out using deep recurrent learning. Now, from the variance output, the abnormality can be identified and resolved. The observed data is combined with previous and healthy function examination data to identify the variances. This combined analysis exploits deep recurrent learning by tuning the analysis layer based on variance. The output makes the progression report concerning the situation of the patient’s disease based on the variance between the normal and abnormal intrinsic neural data connectivity. If the progression is abnormal, further steps are taken to reduce the abnormalities in the patient’s disease report. Figure 14 compares verification time for implemented SPRM and existing MMQA, MGR-ND, and AI-WC for different data inputs and patterns.

Table 1 and Table 2 summarise the comparative analysis of results obtained from the implemented SPRM, and existing MMQA, MGR-ND, and AI-WC. The above results are presented by observing the cumulative progression of data inputs and the patterns from the inputs. Considering the cumulative mean value of the existing methods, the proposed method is validated in terms of ratio.

Summary: The proposed method achieves 13.15% high accuracy, 11.84% high precision, and 8.44% high pattern verification. It reduces the variance and verification time by 12.6% and 10.39%, respectively.

Summary: The proposed method achieves 16.77% high accuracy, 10.55% high precision, and 7.69% high pattern verification. It reduces the variance and verification time by 12.08% and 12.02%, respectively.

## 5. Conclusions

This article introduces an SPRM for identifying neurodegenerative disease progression. The progression is identified using clinical and patient-observed data across multiple instances. The data correlation is based on different patterns exhibited by the input data and is combined for unknown patterns and dissimilarity analysis. The connectivity and variance metrics are validated in this analysis to prevent observation function overflows. The process is extended using deep recurrent learning to identify consecutive sequential abnormalities. The identified abnormalities are validated for the intrinsic data connectivity for progress estimation. The accuracy and precision features are consistently retained from successive iterations by mitigating the abnormalities. Further data analysis is resolved through the same kind of process; therefore, the healthy (previous) and the observation instance data are jointly used for analysis to prevent variance verification. This guides the identification of new unclassified patterns; this unclassified data is normalised using precise data computation for which the new variance is estimated. The unidentified instances generate a chance of causing variations that are suppressed by training from the previous consecutive intervals. The difference between successive variance pronounces the disease progression, correlated to the clinical values. The proposed method achieves 13.15% high accuracy, 11.84% high precision, and 8.44% high pattern verification. It reduces the variance and verification time by 12.6% and 10.39%, respectively. The implemented SPRM has significantly improved the accuracy and efficiency of NDD diagnosis, ultimately leading to better patient outcomes. Despite significant achievements in disease progression identification, the proposed method lags in identifying and slagging missing data. This issue may generate high variances across different observation intervals that impact the accuracy. Therefore, a modified missing value substitution method can be proposed to extend the current work. This work will either reduce the errors or identify the error-causing sequences.

## Figures and Tables

**Table 1:** Summary of data inputs.

Metrics	MMQA	MGR-ND	AI-WC	SPRM
Accuracy	82.44	86.91	90.02	93.034
Precision	0.784	0.841	0.895	0.9584
Pattern Verification	7	12	21	27
Variance	0.232	0.187	0.108	0.0497
Verification Time (s)	6.16	5.02	3.32	1.821

**Table 2:** Summary of patterns.

Metrics	MMQA	MGR-ND	AI-WC	SPRM
Accuracy	81.46	83.65	90.79	93.683
Precision	0.788	0.849	0.904	0.9525
Pattern Verification	8	13	21	26
Variance	0.236	0.174	0.112	0.0532
Verification Time (s)	6.12	5.08	3.24	1.343

## Data Availability

We have provided data web link in ref. [46] and cited in text.
